# Clinical Characteristics and Remission Monitoring of 6q24-Related Transient Neonatal Diabetes

**DOI:** 10.1155/pedi/3624339

**Published:** 2024-11-26

**Authors:** Michael E. McCullough, Lisa R. Letourneau-Freiberg, Tiana L. Bowden, Balamurugan Kandasamy, Angela Ray, Kristen Wroblewski, Daniela del Gaudio, Deborah J. G. Mackay, Louis H. Philipson, Siri Atma W. Greeley

**Affiliations:** ^1^Section of Adult and Pediatric Endocrinology, Diabetes and Metabolism and Kovler Diabetes Center, University of Chicago, Chicago, Illinois, USA; ^2^Department of Hospital Medicine, Children's Hospital Los Angeles, Los Angeles, California, USA; ^3^Department of Public Health Sciences, University of Chicago, Chicago, Illinois, USA; ^4^Department of Human Genetics, University of Chicago, Chicago, Illinois, USA; ^5^Division of Human Genetics, University of Southampton, Southampton, UK

## Abstract

**Introduction:** Transient neonatal diabetes mellitus (TNDM) is a heterogeneous subtype of neonatal diabetes that usually presents within the first days or weeks of life, spontaneously remits in infancy, but can recur in childhood or adolescence as a permanent form of diabetes. Approximately 70% of TNDM cases are due to overexpression of genes at chromosome 6q24 (6q24-TNDM) caused by one of three potential mechanisms: paternal uniparental disomy (pUPD6), paternal duplication, or hypomethylation of the maternal allele. Our aim was to further elucidate the clinical characteristics of a relatively large group of individuals with this rare condition.

**Methods:** Participants with a genetically confirmed diagnosis of 6q24-TNDM were identified through the University of Chicago Monogenic Diabetes Registry. Some participants had testing done on a clinical basis, with the remainder having received research-based genetic testing. Clinical information was extracted from survey responses and medical records.

**Results:** There were 33 participants with 6q24-TNDM (58% were male). Eight (24%) had hypomethylation of the maternal allele, seven (21%) had paternal duplication, 17 (52%) had pUPD6, and one individual had 6q24 hypomethylation of unknown etiology. The median age of initial diabetes presentation was 2 days (*n* = 33). Remission occurred at a median age of 3 months (*n* = 28). The median age of relapse was 14 years (range 12–31 years, *n* = 9). The majority (71%) of participants were born small for gestational age and 32% of participants were born before 37 weeks gestation. The most common extra-pancreatic features were umbilical hernia (22%, *n* = 6/27), macroglossia (56%, *n* = 15/27), and speech pathologies (36%, *n* = 10/28). No significant differences in clinical characteristics were identified across the three genetic etiologies (pUPD6, paternal duplication, maternal hypomethylation).

**Conclusions:** Clinical characteristics were not different across underlying genetic mechanism groups, suggesting that genetic testing is required to definitively determine the mechanism and diagnosis of 6q24-TNDM. Clarification of the specific underlying mechanism is strongly encouraged to clarify recurrence risk, but whether these subcategories may have other clinically relevant differences remains to be elucidated. Early assessment for speech therapy should be considered for this patient population. We recommend that patients in remission be equipped to check blood glucose levels as needed, such as during illness, and should continue seeing a diabetes provider at least occasionally, especially around the time of puberty and thereafter.

## 1. Introduction

Neonatal diabetes, also referred to as congenital diabetes, is characterized by elevated blood glucose levels within the first 6–12 months of life. Neonatal diabetes is rare, with an approximate incidence rate of 1 in 100,000 births, and can either be permanent or transient [1, 2].

Transient neonatal diabetes mellitus (TNDM) is a heterogeneous subtype of neonatal diabetes that usually presents within the first days or weeks of life, spontaneously remits in infancy, but can recur in childhood or adolescence as a permanent form of diabetes. Approximately 70% of TNDM cases are due to overexpression of two maternally imprinted genes (*PLAGL1* and *HYMAI*) at chromosome 6q24 [1, 2]. Overexpression of these genes can be caused by three underlying genetic mechanisms: (1) uniparental paternal disomy of chromosome 6 (pUPD6) in which there are two copies of 6q24, both paternally inherited; (2) paternal duplication of the 6q24 allele in which there are three copies of 6q24, two paternally inherited and one maternally inherited; (3) maternal hypomethylation of the differentially methylated region (DMR) at 6q24 resulting in a silencing defect of the maternal allele [3].

Clinical characteristics and phenotypic features have been shown to differ among 6q24-TNDM patients, both as a whole as well as within each genetic subcategory [4]. However, our understanding remains limited and is challenged by the rarity of 6q24-TNDM. Moreover, recommendations for blood glucose monitoring and other disease management behaviors during the remission period of 6q24-TNDM patients are not clearly defined. Previous studies have not evaluated these behaviors during the remission period.

Therefore, our aim was to further elucidate clinical characteristics and remission monitoring behaviors of a relatively large group of individuals with 6q24-TNDM.

## 2. Materials and Methods

### 2.1. Participants/Data Collection

Thirty-three participants with a genetically confirmed diagnosis of 6q24-TNDM were identified through the University of Chicago Monogenic Diabetes Registry. Participants enrolled in the registry complete a baseline survey that includes questions about demographics, diagnosis history, birth history, clinical history, other medical problems, and family history. A brief follow-up survey is then completed annually about changes in the status of their diabetes, medications, other medical problems, and changes in family history. Participant files are supplemented with medical records and genetic testing information. Data included in this study were extracted from participant REDCap files and medical records. This study was approved by the UChicago Institutional Review Board (UChicago IRB# 15617B, 6858). Written informed consent was obtained from all participants.

A subset of 13 participants responded to inquiries to complete a one-on-one phone interview: two adult participants and the parents of 11 participants. Interviews were semi-structured with a specific focus on blood glucose monitoring, self-reported glycemia during illness, and management by a healthcare provider. The questions were in reference to the remission period, so participants who had relapsed were asked to recall characteristics from their remission.

### 2.2. Genetic Testing Methodology

Research-based genetic testing was performed primarily at the University of Chicago Genetics Services Laboratory (UCGSL) and Wessex Regional Genetics Laboratory (University of Southampton, UK). Sixteen participants had genetic testing completed solely at UCGSL. Four participants had genetic tested completed solely at Wessex. Seven participants had genetic testing completed at both Wessex and UCGSL. Six participants had genetic testing completed at other laboratories.

Genomic DNA was isolated from saliva or blood samples using the Oragene OG-300 noninvasive saliva sampling kit (DNA Genotek Inc., Ottawa, ON, Canada) or the PureGene DNA isolation kit (Qiagen Inc, Valencia, CA, USA) according to the manufacturer's instructions. Methylation-specific polymerase chain reaction (PCR) or methylation-specific multiplex ligation-dependent probe amplification (MS-MLPA) was used to identify hypomethylation of the 6q24 locus. Methylation-specific PCR [5] utilized the divergent sequence changes deriving from bisulfite treatment of differentially methylated DNA, yielding differently sized products in a ratio reflecting that of the starting material. Duplex reactions contained two overlapping forward primers with divergent sequences overlying multiple CpG dinucleotides and one fluorescently labeled reverse primer not overlapping CpG dinucleotides and, therefore, nonselective for the methylation status of DNA. PCR amplification with limited cycles generated maternal and paternal alleles in a ratio reflecting that of the source DNA. MS-MLPA was performed using the SALSA MS-MLPA kit ME033 (MRC-Holland, Amsterdam, The Netherlands) designed to include probes covering the *PLAGL1* gene as well as portions of the *INS*, *KCNJ11*, and *ZFP57* genes. Three of the *PLAGL1* probes contain a *Hha*I site, allowing assessment of the methylation status of the TNDM DMR CpG island. The MS-MLPA assay and data analyses were performed according to the manufacturer's protocol and as previously described (PMID: 26894574). Briefly, probes were simultaneously hybridized to regions throughout the 6q24 region and then exposed to methylation-specific digestion, followed by PCR amplification of the hybridized regions. The PCR products were resolved on an ABI 3730xl DNA Analyzer (Life Technologies, Grand Island, NY, USA), and data analysis was carried out using GeneMarker V.2.6.0 software (Softgenetics LLC, State College, Pennsylvania, USA). Normalization was performed and the peak heights were compared to a synthetic control. Peak heights outside the range of 0.7–1.3 times the control peak height were considered abnormal, with those above 1.3 representing duplications. Quantification of the methylation status was performed by comparison of the restriction-digested aliquot with the paired undigested aliquot from each sample. When parental samples were available, follow-up microsatellite testing using seven markers (D6S1713, D6S389, D6S1613, D6S1639, D6S435, D6S311, and D6S305) was conducted to delineate UPD6 vs isolated maternal hypomethylation. PCR products were resolved on an ABI 3730xl DNA Analyzer, and alleles were called using GeneMapper V.2.6.0 software. In some participants, parental samples were not available. In this case, analysis was conducted only on participant samples to evaluate for homozygosity at the seven short tandem repeats (STR) that were tested. The participant samples identified to be homozygous at all seven STR tested were classified as likely-UPD6 for this study. One participant had MS-MLPA testing indicating 6q24 hypomethylation (and ruled out the possibility of paternal duplication). However, due to a lack of sufficient DNA samples, follow-up microsatellite analysis was not performed and, therefore, this participant was included in the total counts but was excluded from the comparison tests across groups.

### 2.3. Data Analysis

Information collected from medical records and participant survey responses was managed using REDCap electronic data capture tools hosted at the University of Chicago [6, 7]. Demographic, health information, and 6q24-TNDM-associated clinical features were summarized overall and by group using frequency counts and percentages for categorical variables and median (25%–75%) for continuous variables. Comparisons across groups were performed using Fisher's exact tests for categorical variables and Kruskal–Wallis tests for continuous variables. Stata 17 (StataCorp LLC, College Station, TX, USA) was used for statistical analysis.

## 3. Results

This study includes 33 participants with 6q24-TNDM: 19 (58%) males and 14 (42%) females (Table 1). The genetic etiology for 6q24-related diabetes included eight (24%) with hypomethylation of the maternal allele, seven (21%) with paternal duplication, 17 (52%) with pUPD6, and one individual with 6q24 hypomethylation of unknown etiology (paternal duplication ruled out). For the eight participants with hypomethylation of the maternal allele and one with unknown etiology, five had normal ZFP57 analysis. For the remaining four, it was unclear if their genetic testing had included ZFP57 analysis. Two participants with paternal duplications were from the same family.

Participants initially presented with diabetes at a median age of 2 days (*n* = 33). Remission occurred at a median age of 3 months (*n* = 28). Nine participants reported having a relapse of diabetes occurring at a median age of 14 years (range 12–31 years). Of the remaining 24 participants, seven reported that they were still in remission at the time of last contact, and 17 had unknown status. The majority (71%) of participants were born small for gestational age, and 32% of participants were born premature (before 37 weeks gestation). The most common extra-pancreatic features reported by participants were umbilical hernia (22%, *n* = 6/27), macroglossia (56%, *n* = 15/27), and speech pathologies (36%, *n* = 10/28). No significant differences in clinical characteristics were identified across the three underlying genetic mechanisms of 6q24-TNDM (pUPD6, paternal duplication, maternal hypomethylation).

During the neonatal phase of diabetes, none of the participants reported DKA. Moreover, for the 29/33 participants with available clinical details, all were treated with insulin. Six of these participants were also treated with sulfonylureas, with one discontinuing insulin altogether (reported previously [8]) and one other discontinuing the sulfonylurea after no change in c-peptide was observed.

**Figure 1:**
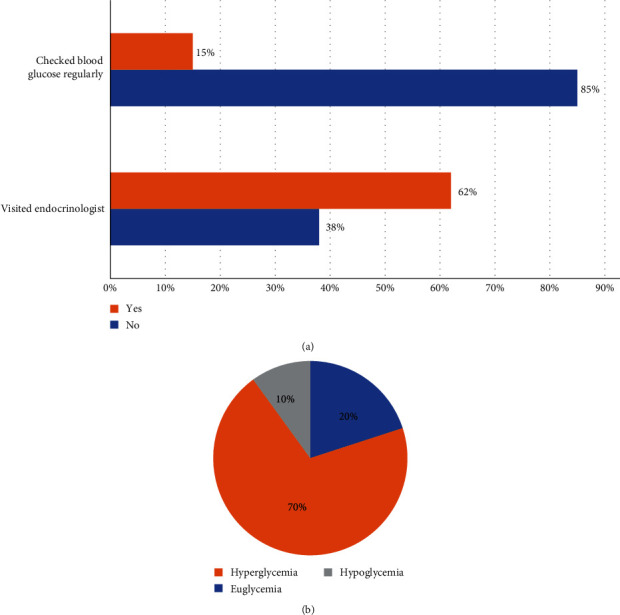
(a) Clinical monitoring during remission period and (b) participant reported glycemic state during times of illness while in diabetes remission.

Thirteen of the participants completed telephone interviews about their diabetes remission phase. At the time of the interviews, participant ages ranged from 1 to 36 years. Eleven participants were still in remission (age 1–10 years), and two had relapsed (ages 21 and 36 years). The two participants who had relapsed answered questions about their past remission phase. Two participants (15%) reported monitoring glucose regularly while in diabetes remission, and eight participants (62%) reported visiting an endocrinologist during the remission period (Figure 1a). Participants were also asked about glycemic response during illness: of the 10 participants who responded, seven (70%) reported euglycemia, two (20%) reported hyperglycemia and one (10%) reported hypoglycemia (Figure 1b). No persistent hypoglycemia was reported by participants during the remission period.

## 4. Discussion

This study describes the genetic and clinical features of 33 research participants with a diagnosis of 6q24-TNDM enrolled in the University of Chicago Monogenic Diabetes Registry. Clinical characteristics were not different across genetic subcategories. Testing to determine the specific genetic subcategory is required to clarify familial recurrence risk, but it remains uncertain if these subcategories may have important clinical differences in treatment response or other long-term associated features. We, therefore, recommend that all cases undergo sufficient testing to definitively determine the underlying genetic etiology of 6q24-TNDM. This is particularly pertinent for 6q24 paternal duplication TNDM patients, who will have a 50% chance of transmitting the duplication to their offspring. Information and resources for obtaining research-based genetic testing can be found at monogenicdiabetes.uchicago.edu or by contacting the University of Chicago Monogenic Diabetes Registry.

We confirmed that this cohort of patients is likely to be small for gestational age (71%) and have a significantly higher incidence of premature birth (32%) compared to the general US population (10.5% in the United States in 2021) [9]. These patients commonly have umbilical hernia and macroglossia. Speech pathologies were also reported in a considerable number of participants. Interestingly, only 40% of those who reported speech pathologies had been diagnosed clinically with macroglossia. Nonetheless, since macroglossia relies on subjective clinical judgment, it is possible that there exists a potential link between tongue overgrowth and the necessity for speech therapy in these instances. We therefore recommend close developmental evaluation, including speech assessment for all individuals with 6q24-TNDM.

The majority of participants reported that they did not routinely monitor their glucose levels during the period of diabetes remission; however, more than half still followed with an endocrinologist. Moreover, 10 participants still checked blood glucose levels during illness, with a third reporting dysglycemia: 2/10 reported hyperglycemia and 1/10 reported hypoglycemia. In this cohort, none of the participants reported persistent hypoglycemia that has been observed in a small fraction of these patients after diabetes remission [10, 11], but rather mild self-limited hypoglycemia that might reflect abnormal regulation of insulin secretion. That 2/10 also reported hyperglycemia reflects previous data suggesting that these individuals retain a robust capacity for insulin production but have defects in glucose-stimulated insulin secretion, which could have effects not only in insulin secretion during hyperglycemia but also the suppression of insulin during hypoglycemia (cite Valerio 2004 and Carmody) [12, 13].

As such, blood glucose monitoring during periods of illness should be considered for 6q24-TNDM patients in remission. More information is still needed on the ideal frequency and method of monitoring; however, based on the information gathered so far, clinicians could consider advising families to have a blood glucose monitoring device (blood glucose meter or continuous glucose monitor) readily available during times of illness and use it to check glucose if the patient is exhibiting symptoms of hyper- or hypoglycemia. Furthermore, during the remission period, clinicians could consider seeing patients annually to check hemoglobin A1c and educate parents on the above-noted remission period monitoring recommendations. These patients often stop seeing a provider and only return when they have already become symptomatic with significant hyperglycemia. So, in educating the patients, it is important to emphasize that they are very likely to have a relapse at some point, either during puberty or later. It is likely that the relapse occurs due to increased demand for insulin production because of increased insulin resistance that can arise with age, especially during puberty. The factors influencing the age of diagnosis could also include frequency of monitoring where some patients may be diagnosed at later ages only because they were not checking blood glucose sooner. We would, therefore, emphasize the importance of returning to see a diabetes provider around puberty and at least annually thereafter.

As reported previously [8], these participants with 6q24-TNDM did not have DKA during the neonatal phase of diabetes. As part of the current data collection, no other participants reported episodes of DKA during either the neonatal or later relapse phase of diabetes. Additionally, as previously described [14], some participants reported a response to sulfonylureas, but the majority of participants appeared to be managed with insulin during the neonatal phase of diabetes. We previously reported one case of 6q24-TNDM that had hyperinsulinism after remission of the diabetes [11]. No other cases in the current data collection reported persistent hypoglycemia.

Although there is still more to learn about 6q24-TNDM, this study provides further insight into this rare condition. In addition to describing neonatal onset characteristics for the different genetic subcategories, we provide rare data about monitoring of blood glucose levels during the remission phase, as well as the age of relapse. Although there were no statistically significant differences in clinical features between genetic subcategories, it is important to note that the number of individuals is relatively low and may be underpowered to detect such differences.

Other relevant limitations include limited clinical information availability for certain participants, recall bias, and self-reported survey data.

In conclusion, genetic testing is required to conclusively determine the diagnosis of 6q24-TNDM, genetic subcategory, and risk of recurrence. Early developmental assessment that includes speech evaluation is recommended for all cases of 6q24-TNDM. These patients could benefit from monitoring their blood glucose levels during illness and should, therefore, maintain contact with a diabetes provider who can prescribe blood glucose monitoring supplies. We further recommend that they continue to be monitored for the relapse of diabetes (such as with HbA1c), especially around the time of puberty and at least annually thereafter. Future studies should further explore characteristics of diabetes relapse in this population in order to clarify optimal treatment strategies that may be amenable to a precision medicine approach, as well as clarifying the risk of diabetes-related or other long-term complications.

## Figures and Tables

**Table 1:** Clinical characteristics of 6q24-TNDM participants.

	Total*⁣*^*∗*^	Paternal duplication	UPD6	Hypomethylation	*p*-Value
Biological sex	—	—	—	—	0.52
* n*	33	7	17	8	—
Female	14 (42%)	3 (43%)	6 (35%)	5 (62.5%)	—
Male	19 (58%)	4 (57%)	11 (65%)	3 (37.5%)	—
Age at last contact (years)	—	—	—	—	0.82
*n*	33	7	17	8	—
Median (IQR)	6 (1–15)	8 (1–27)	8 (1–15)	4 (1.5–16.5)	—
Birthweight (g)	—	—	—	—	0.68
*n*	31	6	17	7	—
Median	2013	1928	2013	2098	—
IQR	1701–2481	1840–2211	170–2481	1446–2325	—
Gestational age (weeks)	—	—	—	—	0.14
* n*	33	7	17	8	—
Median (IQR)	37 (35–39)	37 (34–40)	38 (37–40)	36 (34–38)	—
Age of initial diabetes presentation (days)	—	—	—	—	0.27
*n*	33	7	17	8	—
Median (IQR)	2 (1–7)	1 (1–2)	2 (1–7)	4 (1.5–12)	—
Age of remission (months)	—	—	—	—	0.30
*n*	28	6	13	8	—
Median (IQR)	3 (2–6)	2.25 (2–3)	3 (3–6)	3.5 (1.5–6)	—
Age of relapse (years)	—	—	—	—	0.28
*n*	9	2	5	2	—
Median (IQR)	14 (13–19)	23 (15–31)	13 (12–14)	16 (13–19)	—
Macroglossia	—	—	—	—	0.47
*n* present	15 (56%)	3 (75%)	7 (47%)	5 (71%)	—
Total	27	4	15	7	—
Umbilical hernia	—	—	—	—	0.07
*n* present	6 (22%)	0 (0%)	2 (13%)	4 (57%)	—
Total	27	4	15	7	—
Speech therapy	—	—	—	—	1.00
*n* present	10 (36%)	2 (40%)	5 (33%)	3 (43%)	—
Total	28	5	15	7	—

*⁣* ^*∗*^One participant who could not be definitively classified was included in the total but not within the genetic subcategories.

## Data Availability

Data supporting the findings of this study may be requested from the corresponding author, Siri Atma W. Greeley (sgreeley@uchicago.edu), upon reasonable request.
